# Mediating role of chronic inflammatory airway diseases in the association between volatile organic compounds exposure and depression

**DOI:** 10.1097/MD.0000000000048522

**Published:** 2026-04-24

**Authors:** Jingshan Bai, Qian Huang, Wenqiang Li, Ying Yang, Zhiping Deng, Quan Yuan

**Affiliations:** a Xiongan Xuanwu Hospital, Hebei, China; b Dazhou Dachuan District People’s Hospital (Dazhou Third People’s Hospital), Sichuan, China; c Zigong First People’s Hospital, Sichuan, China.

**Keywords:** chronic inflammatory airway diseases, depression, mediator, NHANES, volatile organic compound

## Abstract

Depression is a prevalent mental disorder that imposes a substantial global burden, and environmental exposure has been increasingly implicated in its etiology. However, the specific mechanisms linking urinary exposure to volatile organic compounds (VOCs) with depression remain poorly understood. A potential association of VOCs with depression prevalence was explored in this study, which also assessed the mediating effect of chronic inflammatory airway diseases (CIAD). This study analyzed data from the National Health and Nutrition Examination Survey from 2011 to 2020, involving 1696 participants. An ultra-high-performance liquid chromatography–tandem mass spectrometry assay was employed to quantify the levels of 16 VOCs in urine samples. We employed weighted multivariable logistic regression models, coupled with mediation analysis, to assess the potential association between urinary concentrations of VOCs and the risk of depression, while also investigating the intermediary pathway involving CIAD. The diagnostic potential of urinary VOCs as biomarkers for depression was further assessed through receiver operating characteristic curve analysis. Screening of the 1696 study subjects indicated a depression prevalence of 11.4%. Notably, 4 urinary VOC metabolites demonstrated a statistically significant positive association with the disorder (odds ratio > 1, *P* < .05). The dose–response relationship between them was also statistically significant. Mediation analysis indicated that CIAD mediated 7.3% of the total effect of VOC exposure on depression (*P* < .05). Contact with certain VOCs has been linked to an elevated incidence of depression disorder. CIAD plays a mediating role in the correlation between urinary VOCs and depression.

## 1. Introduction

Depression is a common mental illness characterized by persistent low mood, lack of energy, and loss of interest or pleasure in activities.^[1]^ Amidst societal advancements, the incidence of depressive disorders has demonstrated a marked escalation globally. A significant intensification of worldwide morbidity metrics is anticipated, potentially driving concurrent rises in non-disease-specific fatality indices.^[2,3]^ Severe depression increases the risk of other diseases and suicide.^[4]^ Depression affects nearly one-third of a billion individuals globally, demonstrating epidemiological dominance as the foremost contributor to mental health disorders.^[5]^ Emerging epidemiological patterns reveal a worldwide surge in psychiatric morbidity, with incidence rates demonstrating consistent annual growth. The annual financial burden of psychiatric care in Europe eclipses national investments in cancer management, heart disease treatment, and diabetic care.^[6]^ There have been numerous studies on the causes of depression. The pathogenesis of depressive disorders reveals significant contributions from ambient environmental stressors and integrated neuroendocrine dysregulation.^[7]^

Accelerated industrial expansion has established environmental contamination as the predominant driver of pathogenesis in human populations. Volatile organic compounds (VOCs) comprise a class of carbon-based substances characterized by reduced molecular weight and high vapor pressure under ambient conditions.^[8,9]^ VOCs are pervasive environmental contaminants originating from both natural sources and anthropogenic activities such as industrial production, transportation, and household product use.^[10]^ The prevalence of VOC-containing consumer goods, coupled with the inherent volatility of these compounds, leads to elevated levels of benzene, toluene, and xylene in occupied indoor environments.^[11]^ The predominant exposure routes to these volatile organic compounds comprise respiratory inhalation, dermal absorption, and oral intake via contaminated media.^[12]^ Clinical studies establish VOC exposure as a significant risk factor for disorders affecting the nervous, pulmonary, and circulatory systems, and carcinogenesis.^[13,14]^ Recently, the results of a study showed that the exposure levels of various urinary VOC metabolites were significantly and positively correlated with the risk of depression.^[15]^ Through multiple linear and logistic regression models, the study found that among the 16 analyzed VOCs, 10 had a positive association with depression, indicating that both single and mixed VOC exposures may have a negative impact on mental health.^[15]^

Chronic inflammatory airway diseases (CIAD), mainly including asthma and chronic obstructive pulmonary disease (COPD), are among the leading causes of morbidity and mortality globally, posing a serious public health challenge.^[16]^ The pathophysiological processes of these diseases are complex, involving the interaction between genetics and various environmental factors, with chronic airway inflammation being their core feature.^[17]^ Increasing evidence shows that environmental VOC exposure is closely related to the risk of childhood asthma.^[18]^ Urinary VOCs are not only associated with the risk of asthma but also have a direct connection with the pathophysiological indicators of the disease, such as lung function and airway inflammation.^[19]^ Similar to asthma, VOC exposure has also been proven to be an important risk factor for COPD. An investigation of 782 subjects demonstrated that 7 urinary VOCs exhibited a significant correlation with elevated COPD risk, accompanied by a clear dose–response gradient.^[20]^ Epidemiological evidence consistently demonstrates significantly elevated prevalence rates of affective disorders among individuals with chronic inflammatory airway conditions (CIAD) compared to general population controls.^[21,22]^ A cohort study of COPD patients found that patients who reported difficulty in affording medications due to cost issues had aggravated anxiety and depression symptoms and a poorer quality of life.^[23]^

This investigation utilizes nationally representative data from the National Health and Nutrition Examination Survey (NHANES) to examine potential associations between VOC exposure and depression. In addition, despite the recognition of this complex interaction among CIAD, VOCs, and depression, the exact role of CIAD as a mediator between VOCs and depression has not been fully explored. Most previous studies have focused on the direct effects of CIAD or VOCs on depressive symptoms, without fully considering the potential mediating role of CIAD. Therefore, this study explores the mediating role of CIAD in the relationship between VOCs and depression.

## 2. Methods and analysis

### 2.1. Study population

This population-based analysis employed 5 consecutive cycles (2011–2020) of the NHANES, leveraging its cross-sectional design to establish temporal associations. The NHANES constitutes a nationally representative surveillance program spearheaded by the National Center for Health Statistics to systematically evaluate health and nutritional dynamics within American communities. This nationally representative survey employs a multistage probability sampling design to capture comprehensive health metrics across the U.S. population, integrating structured interviews, standardized physical examinations, and laboratory-based biomarker analyses. This study adhered to the Strengthening the Reporting of Observational Studies in Epidemiology reporting guidelines and involved a secondary analysis of de-identified, publicly available data from NHANES.

**Figure 1.**
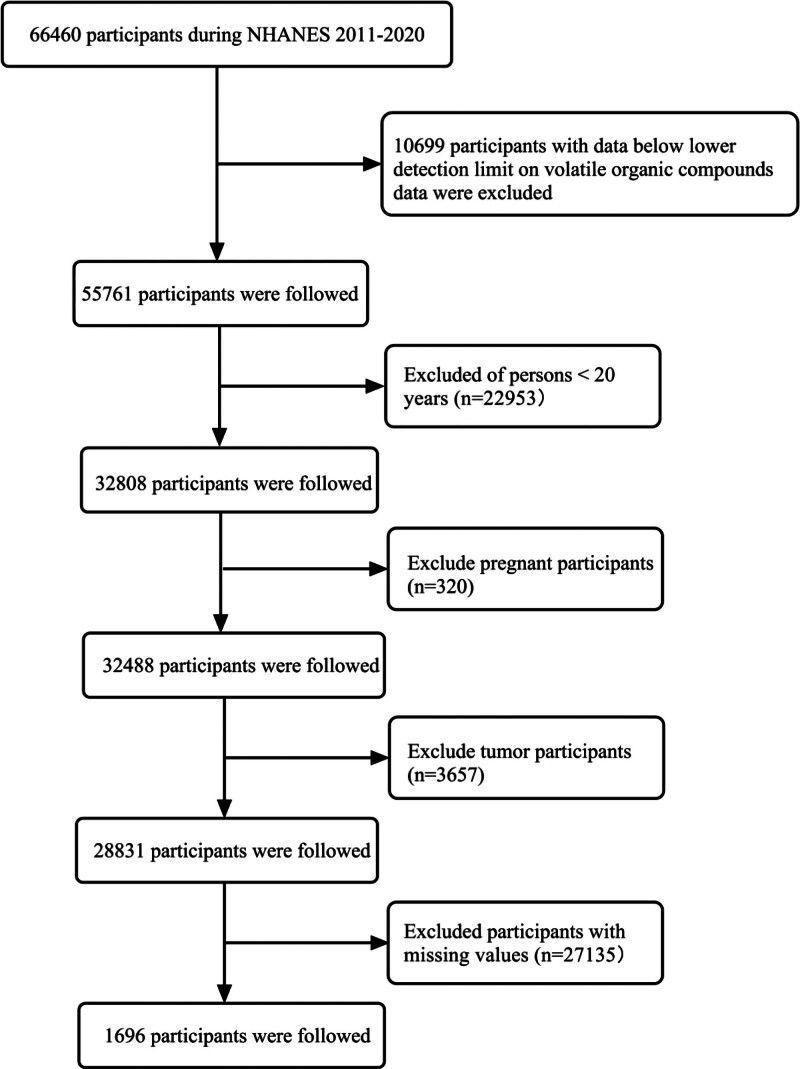
Flowchart of participant selection.

The analytical cohort comprised 66,460 individuals derived from 5 consecutive NHANES cycles. Eligibility criteria required available urinary VOC profiles and a minimum age of 20 years, yielding an analytical sample of 32,808 after excluding 10,699 cases with incomplete biomarker data. In addition, 320 pregnant participants and 3657 participants with tumors were excluded. To obtain more reliable results, 27,135 participants with missing covariates were also excluded. The final analytical cohort comprised 1696 study participants (Fig. 1).

### 2.2. Urine VOCs determination

Urine specimens were obtained under standardized collection protocols to preserve the analytical validity and biological relevance of VOC metabolite quantifications. Urine specimen acquisition was systematically aligned with each participant’s arrival at the Mobile Examination Center, establishing a standardized temporal window for biospecimen collection relative to the clinical visit schedule. The protocol stipulated strict storage procedures. Samples were required to be transported and stored under refrigeration or frozen at ‐20 °C before being transferred to −70 °C for long-term storage. Moreover, the number of freeze–thaw cycles was strictly limited to no more than 5 times. The U.S. NHANES employs ultra-high-performance liquid chromatography–electrospray tandem mass spectrometry to quantify volatile organic compound metabolites in human urine.^[20]^ Ultra-high-performance liquid chromatography–electrospray tandem mass spectrometry enables highly sensitive and specific quantification of volatile organic compounds and their metabolic derivatives. This technique demonstrates superior performance characterized by rapid analytical velocity, elevated throughput, exceptional detection sensitivity, robust multiplexing capacity for parallel analyte quantification, mild ionization efficiency, and precise internal standard-based calibration accuracy. Urinary VOC measurements falling below the limit of detection were omitted in this analysis to ascertain actual concentration levels within the sampled population. The final analytical panel comprised 16 distinct volatile organic compounds quantified in urine specimens. Table S1, Supplemental Digital Content, https://links.lww.com/MD/R775 summarizes the quantified analytes, their designated code nomenclature, and associated precursor compounds.

### 2.3. Assessment of CIAD and cardiovascular disease (CVD)

In the present study, CIAD encompasses self-reported cases of asthma, chronic bronchitis, and COPD. The questionnaire inquired whether participants had ever received a diagnosis of asthma, chronic bronchitis, or COPD from a doctor or other healthcare providers. Participants who responded affirmatively were regarded as having this disease.^[24]^

CVD was defined as any previously diagnosed congestive heart failure, coronary heart disease, angina, or myocardial infarction reported by participants.^[25]^ Information used to evaluate cardiovascular health was derived from the cardiovascular disease and health section of the questionnaire, which comprised participant-level interview responses. These interviews were conducted in participants’ homes by trained interviewers using a computer-assisted personal interviewing system. This standardized and structured approach minimized potential sources of bias and enhanced the reliability of self-reported data.

### 2.4. Assessment of depression

The study employed the Patient Health Questionnaire-9, a 9-item instrument with established sensitivity and specificity for detecting depression-related clinical manifestations.^[26]^ The response options for the 9 questions regarding symptoms include “Not at all,” “Several days,” “More than half the days,” and “Nearly every day,” with each option corresponding to a score ranging from 0 to 3 points. The total score ranges from 0 to 27 points. In this study, a total Patient Health Questionnaire-9 score of ≥10 was defined as depression.

### 2.5. Covariates

Covariates extracted from the survey questionnaire encompassed the following categorical variables: age (≤44, 45–64, ≥65), sex (male/female), marital status (never married, married/cohabiting, widowed/divorced/separated), race (Non-Hispanic White, Non-Hispanic Black, Mexican American, other groups), education leve (<high school, high school diploma, >high school), hypertension (yes/no), diabetes mellitus (yes/borderline/no), body mass index (BMI) (<25, 25–30, >30), and poverty income ratio (PIR) (<1.3, 1.3–3.5, >3.5). PIR is defined as the ratio of family income to a specific poverty threshold for the survey year, with the denominator determined by the poverty guidelines set by the Department of Health and Human Services. Diagnoses of diabetes and hypertension were self-reported by the participants through questionnaires.^[27]^

### 2.6. Statistical analyses

In this study, the analysis was weighted with the appropriate sample weights supplied by NHANES to address the complex sampling design of NHANES. Continuous measures are presented as mean ± standard deviation (SD), and categorical data are summarized using frequency counts and percentages. Between-group comparisons were evaluated through application of the *T* test to continuous variables and the Chi-square test to categorical variables. The present analysis utilized Pearson correlation testing to evaluate potential co-exposure patterns of VOCs. Multivariate logistic regression was used to determine the relationship between VOCs and depression. Within the established modeling framework, separate regression analyses were conducted for each VOC following concentration unit adjustments to preliminarily examine associations between VOC exposure metrics and depression incidence probabilities. To characterize potential nonlinear relationships, dose-response associations between VOC exposure levels and depression risk were evaluated using restricted cubic spline regression modeling. Furthermore, mediation analysis was employed to assess the mediating role of CIAD and CVD in the relationship between urinary VOCs and depression. The investigation into the relationships between VOCs and depression mixtures was conducted using weighted quantile sum (WQS) regression. The diagnostic performance of candidate urinary VOC biomarkers for depression prediction was evaluated using receiver operating characteristic (ROC) curve analysis.

## 3. Results

### 3.1. The baseline characteristics of included participants

Table 1 summarizes the demographic profile of the study cohort across the 2011 to 2020 survey period. A total of 11.43% (194/1696) of the participants were diagnosed with depression. The depression group exhibited a greater prevalence of female participants relative to the nondepression cohort. There were also significant differences between the 2 groups in terms of PIR, educational level, diabetes, hypertension, and blood creatinine levels. However, there were no statistically significant differences in the distribution of age, race, marital status, and BMI between the 2 groups (*P* > .05).

**Table 1:** Characteristics of the study population.

Variable	Total	Depression		*P* value
		No	Yes	
Sex, n (%)				<.001
Female	790 (46.37)	669 (44.19)	121 (63.14)	
Male	906 (53.63)	833 (55.81)	73 (36.86)	
Age, n (%)				.23
20–44 years	916 (57.58)	813 (58.30)	103 (52.09)	
45–64 years	576 (32.85)	501 (31.80)	75 (40.94)	
≥65 years	204 (9.57)	188 (9.90)	16 (6.97)	
Marital status, n (%)				.1
Never married	419 (22.83)	367 (22.52)	52 (25.24)	
Married/living with partner	919 (56.93)	831 (58.20)	88 (47.16)	
Widowed/divorced/separated	358 (20.24)	304 (19.28)	54 (27.60)	
Race, n (%)				.22
Non-Hispanic White	592 (60.40)	521 (60.74)	71 (57.75)	
Non-Hispanic Black	551 (17.13)	494 (17.31)	57 (15.73)	
Mexican American	225 (9.71)	199 (9.87)	26 (8.54)	
Other races	328 (12.76)	288 (12.08)	40 (17.99)	
PIR, n (%)				.002
< 1.3	652 (28.88)	554 (27.43)	98 (39.99)	
1.3–3.5	648 (41.53)	577 (41.37)	71 (42.75)	
>3.5	396 (29.60)	371 (31.20)	25 (17.25)	
BMI, kg/m^2^, n (%)				.36
<25	441 (23.46)	393 (23.11)	48 (26.14)	
25–30	475 (28.16)	436 (28.94)	39 (22.18)	
>30	780 (48.38)	673 (47.95)	107 (51.67)	
Educational level, n (%)				<.0001
<High school	373 (17.07)	306 (15.76)	67 (27.18)	
High school diploma	444 (28.58)	390 (27.95)	54 (33.44)	
>High school	879 (54.35)	806 (56.30)	73 (39.38)	
Hypertension, n (%)				.003
No	1116 (69.15)	1011 (70.59)	105 (58.07)	
Yes	580 (30.85)	491 (29.41)	89 (41.93)	
Diabetes mellitus, n (%)				<.001
Borderline	50 (2.20)	44 (2.13)	6 (2.76)	
No	1452 (88.79)	1299 (90.04)	153 (79.14)	
Yes	194 (9.01)	159 (7.83)	35 (18.10)	
Serum creatinine, mean (SD)	0.87 (0.01)	0.88 (0.01)	0.84 (0.01)	.01

BMI = body mass index, PIR = poverty income ratio, SD = standard deviation.

Excluding URXAAM, URXATC, URXDHB, URXHP2, URX2MH, and URX34M, the depression group exhibited significantly elevated concentrations of 10 urinary VOCs compared to the nondepression cohort (*P* < .05) (Table 2).

**Table 2:** The VOCs characteristics of the study population.

Variable	Total	Depression		*P* value
		No	Yes	
URXAAM	193.86 (6.86)	189.35 (6.49)	228.50 (23.24)	.09
URXAMC	535.56 (25.73)	507.64 (26.55)	750.32 (69.54)	.001
URXATC	264.66 (6.74)	260.11 (7.16)	299.57 (22.36)	.09
URXCEM	293.07 (12.07)	282.38 (13.09)	375.33 (30.20)	.01
URXCYM	131.96 (7.48)	122.61 (7.11)	203.90 (25.64)	.002
URXDHB	612.26 (11.17)	609.38 (12.00)	634.38 (40.08)	.56
URXGAM	26.03 (0.75)	25.26 (0.71)	31.92 (2.75)	.02
URXHEM	3.73 (0.15)	3.55 (0.16)	5.06 (0.54)	.01
URXHPM	1131.64 (68.30)	1085.45 (74.57)	1486.86 (155.34)	.02
URXHP2	111.53 (6.25)	111.08 (6.83)	115.02 (13.26)	.79
URXPMM	1102.83 (51.60)	1037.02 (55.95)	1609.00 (164.99)	.002
URXMAD	336.32 (9.78)	323.31 (10.17)	436.38 (39.49)	.01
URX2MH	128.00 (9.00)	125.51 (9.88)	147.14 (14.48)	.2
URX34M	952.12 (109.46)	940.26 (123.31)	1043.33 (90.25)	.5
URXMB3	29.88 (1.65)	28.07 (1.82)	43.83 (4.57)	.002
URXPHG	460.89 (10.81)	448.21 (10.44)	558.36 (37.81)	.005

### 3.2. Correlation analysis among VOCs

**Figure 2.**
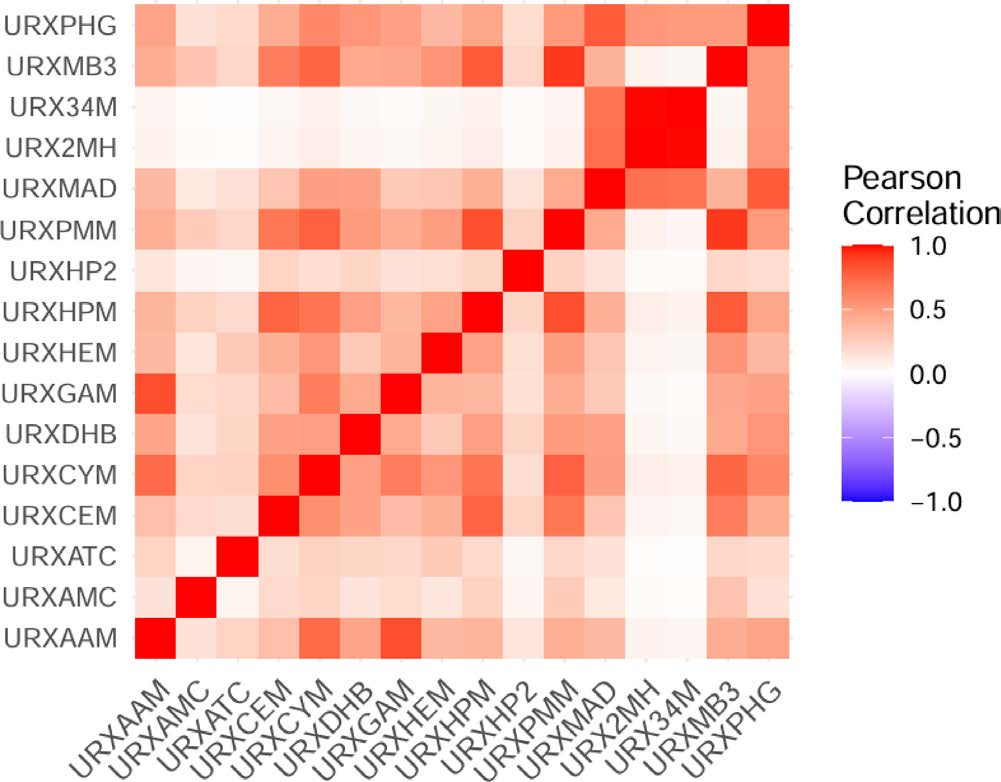
Heatmap of VOCs correlations. VOCs = volatile organic compounds.

Pearson correlation analysis was employed to assess potential co-exposure patterns of VOCs. Figure 2 demonstrates significant positive correlations across multiple volatile organic compounds. Specifically, we found strong correlations between URX34M and URX2MH (*R* = 0.99), URXMB3 and URXPMM (*R* = 0.92), URXGAM and URXAAM (*R* = 0.85), and URXPMM and URXHPM (*R* = 0.85).

### 3.3. Multivariable logistics analysis of the association between VOCs exposure and depression

Weighted multiple logistic regression modeling was employed to evaluate the association between exposure to urinary VOCs and depression risk. Multivariable-adjusted regression models controlling for sex, age, marital status, race, poverty-income ratio, BMI, educational attainment, hypertension, diabetes mellitus, and serum creatinine levels revealed significant positive associations between urinary concentrations of URXCYM, URXGAM, URXMAD, and URXPHG with depression risk (Table 3). The other 12 components of VOCs (URXAAM, URXAMC, URXATC, URXCEM, URXDHB, URXHEM, URXHPM, URXHP2, URXPMM, URX2MH, URX34M, and URXMB3) did not show any significant effects. Although some odds ratios appear numerically close to 1.0, these estimates correspond to very small unit increases in VOC concentrations. When exposure increments are scaled, the associated increases in depression risk become more apparent.

**Table 3:** Association between VOCs and depression in logistic regression.

VOCs (urine, ng/mL)	OR (95% CI)	*P*
URXAAM	1.001 (1.000–1.002)	.154
URXAMC	1.000 (1.000–1.000)	.206
URXATC	1.000 (0.999–1.001)	.915
URXCEM	1.000 (1.000–1.001)	.105
URXCYM	1.001 (1.000–1.002)	.009
URXDHB	1.000 (0.999–1.001)	.58
URXGAM	1.010 (1.001–1.018)	.032
URXHEM	1.030 (0.993–1.067)	.11
URXHPM	1.000 (1.000–1.000)	.334
URXHP2	1.000 (0.999–1.001)	.545
URXPMM	1.000 (1.000–1.000)	.069
URXMAD	1.001 (1.000–1.001)	.007
URX2MH	1.000 (1.000–1.000)	.159
URX34M	1.000 (1.000,1.000)	.275
URXMB3	1.003 (0.999–1.008)	.145
URXPHG	1.001 (1.000–1.001)	.011

OR = odds ratio.

As shown in Table 4, for every one-unit (10^‐2^ ng/mL) increase in URXGAM, the risk of depression increases by 2.60 (1.09, 6.18). For every one-unit (10^‐3^ ng/mL) increase in URXCYM, URXMAD, and URXPHG, the risk of depression increases by 3.58 (1.39, 9.24), 2.33 (1.27, 4.27), and 2.26 (1.21, 4.21), respectively.

**Table 4:** Multivariate logistic regression models of 4 VOCs with different unit concentrations.

VOCs	OR (95% CI)	*P*
*URXCYM*	1.001 (1.000–1.002)	.009
URXCYM (per 10 ng/mL)	1.01 (1.00–1.02)	.01
URXCYM (per 100 ng/mL)	1.14 (1.03–1.25)	.01
URXCYM (per 1000 ng/mL)	3.58 (1.39–9.24)	.01
*URXGAM*	1.010 (1.001–1.018)	.032
URXGAM (per 10 ng/mL)	1.10 (1.01–1.20)	.03
URXGAM (per 100 ng/mL)	2.60 (1.09–6.18)	.03
*URXMAD*	1.001 (1.000–1.001)	.007
URXMAD (per 10 ng/mL)	1.01 (1.00–1.01)	.01
URXMAD (per 100 ng/mL)	1.09 (1.02–1.16)	.01
URXMAD (per 1000 ng/mL)	2.33 (1.27–4.27)	.01
*URXPHG*	1.001 (1.000–1.001)	.011
URXPHG (per 10 ng/mL)	1.01 (1.00–1.01)	.01
URXPHG (per 100 ng/mL)	1.08 (1.02–1.15)	.01
URXPHG (per 1000 ng/mL)	2.26 (1.21–4.21)	.01

OR = odds ratio.

### 3.4. Dose–response relationship between VOCs exposure and depression

**Figure 3.**
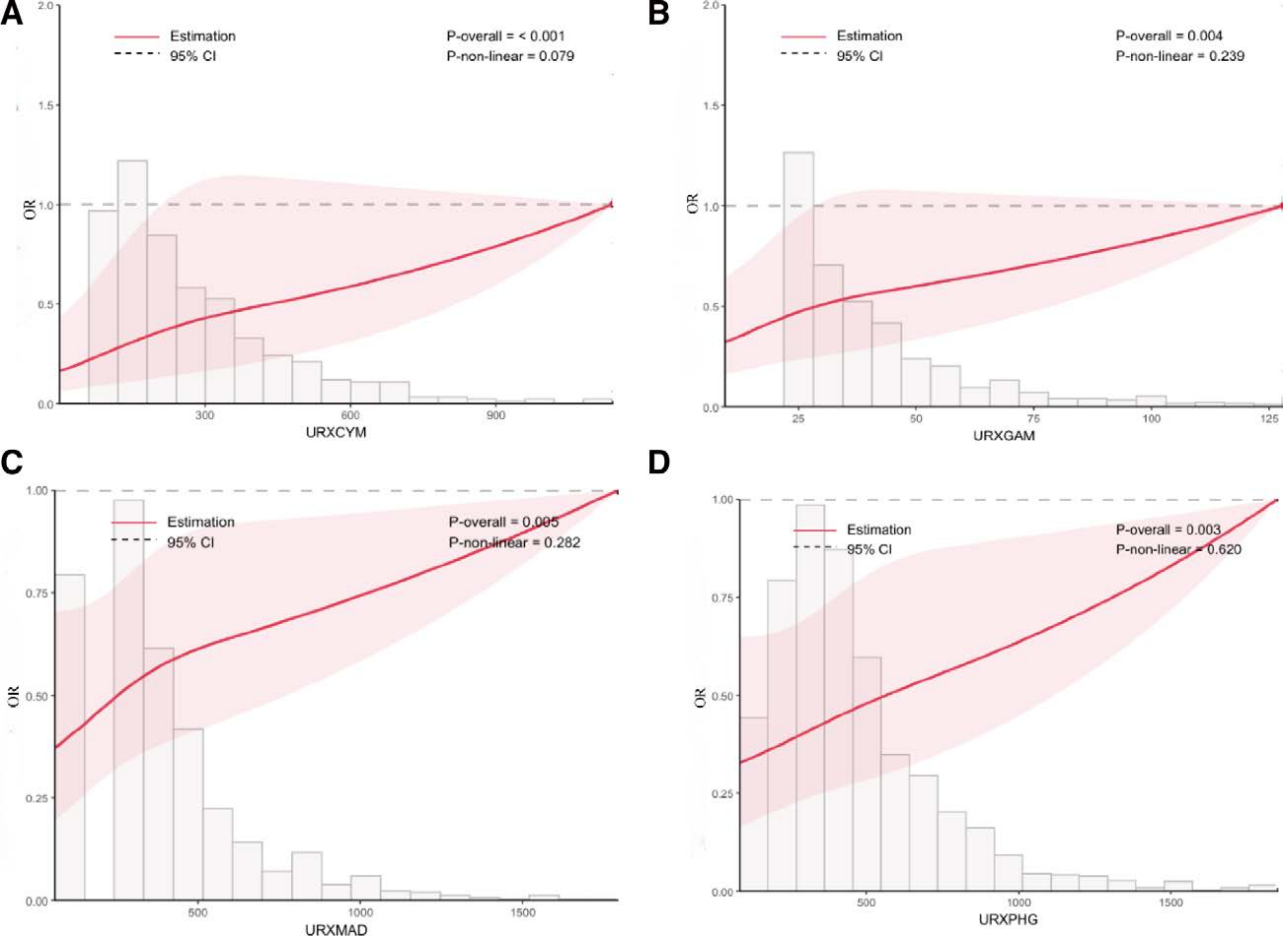
Dose–response curves between 4 urine VOCs and depression. (A) Dose–response curves between URXCYM and depression. (B) Dose–response curves between URXGAM and depression. (C) Dose–response curves between URXMAD and depression. (D) Dose–response curves between URXPHG and depression. VOCs = volatile organic compounds.

To validate the actual dose–response relationship between urinary VOCs and depression, an extensive correlational analysis was performed targeting 4 VOC metabolites demonstrating associations with depression incidence (Fig. 3). A significant dose–response gradient was observed for urinary VOCs levels in relation to depression risk (*P* overall < .05). A linear association was consistently observed between urinary VOCs and depression risk, with no evidence of nonlinear patterns (nl-*P* value > .05).

### 3.5. WQS regression analysis of associations between VOCs exposure and depression

**Figure 4.**
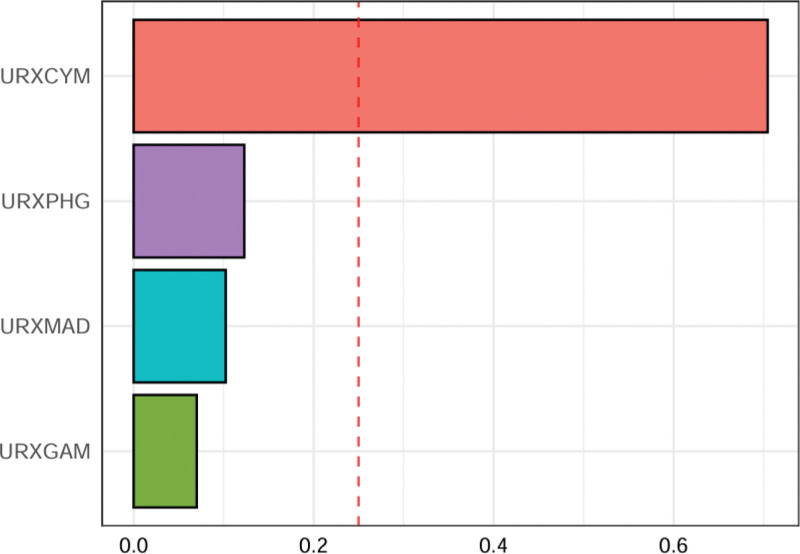
The estimated weights assigned to each VOC contributed to the combined effect on depression across all participants. VOC = volatile organic compound.

WQS regression modeling was applied to examine the relationship between combined VOCs exposure and depression risk (Fig. 4). We observed that URXCYM had the highest weight (70.46%), followed by URXPHG (12.29%) and URXMAD (10.23%). We found that there was a statistically significant positive correlation between exposure to the VOC mixture and the risk of depression. For every one-unit increase in the WQS, the risk of depression increased by 22% (odds ratio: 1.22, 95% CI: 1.01–1.42, *P* = .0392).

### 3.6. Mediation analysis

To examine the associations among CIAD, CVD, VOCs, and depression, a mediation analysis framework was applied to evaluate the intermediary effects of CIAD and CVD on the relationship between VOC exposure and depression risk. Table 5 delineates the direct and mediated pathways linking VOCs to depression, wherein CIAD and CVD operate as causal intermediaries. In summary, CIAD was confirmed as an important mediator in the relationship between VOCs and depression. The contribution rates of these factors to the mediation effect were 7.30%, 7.09%, and 10.50%. However, CVD showed no mediation effect (*P* > .05).

**Table 5:** The mediating effects of CIAD and CVD on the association between VOCs and risk of depression.

Inflammatory factors	Indirect effects	Direct effects	Total effects	Mediated proportion (%)	*P* value
	β (95 CI%)	β (95 CI%)	β (95 CI%)		
CIAD	2.63 × 10^‐6^ (5.74 × 10^‐8^, 0.00)	3.33 × 10^‐5^ (2.00 × 10^‐5^, 0.00)	3.60 × 10^‐5^ (2.29 × 10^‐5^, 0.00)	7.30	.008
CVD	2.99 × 10^‐7^ (6.24 × 10^‐7^, 0.00)	3.58 × 10^‐5^ (2.34 × 10^‐5^, 0.00)	3.61 × 10^‐5^ (2.38 × 10^‐5^, 0.00)	0.83	.65

CIAD = chronic inflammatory airway diseases, CVD = cardiovascular diseases.

### 3.7. ROC curve analysis of urine VOCs in the diagnosis of depression

**Figure 5.**
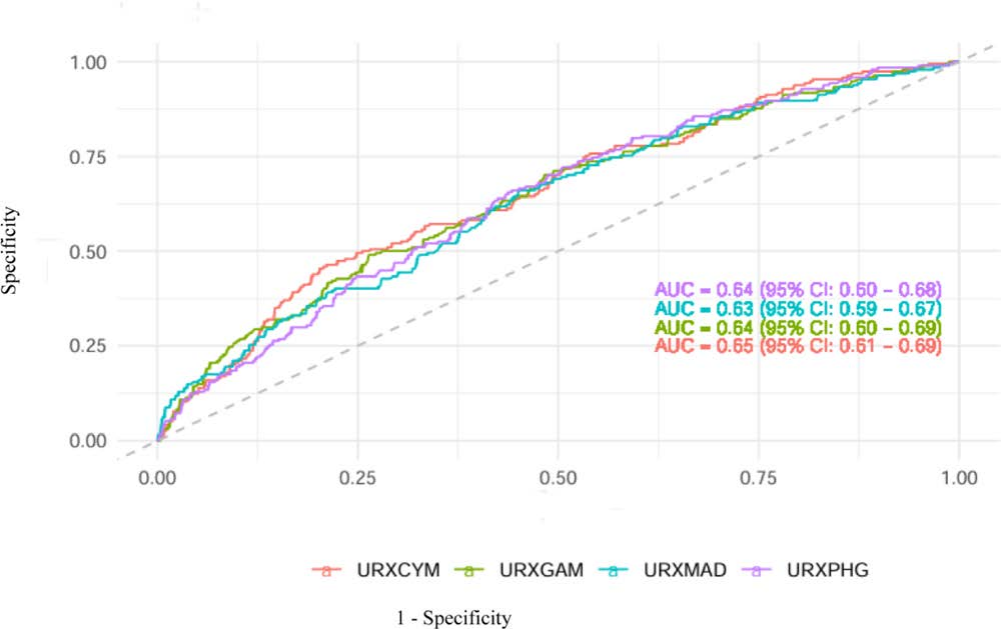
ROC curves of VOCs to diagnose depression. ROC = receiver operating characteristic, VOCs = volatile organic compounds.

The ROC curve analysis was employed to assess the diagnostic performance of urinary VOCs in depression identification, with sensitivity and specificity metrics quantified (Fig. 5). These 4 urinary VOCs showed good predictive ability in the diagnosis of depression. Among them, URXCYM had the best predictive ability (area under the receiver operating characteristic curve = 0.65, 95% CI: 0.61–0.69), which was similar to the result shown in the WQS regression analysis.

## 4. Discussion

This cross-sectional analysis utilized 2011 to 2020 NHANES data to examine associations between urinary VOCs exposure and depression prevalence among U.S. adults. Weighted logistic regression modeling demonstrated significant positive associations between urinary concentrations of URXCYM, URXGAM, URXMAD, URXPHG, and depression risk. CIAD may be an intermediate variable between urinary VOCs and depression. Furthermore, ROC curve analysis demonstrated that urinary VOCs exhibit strong predictive accuracy for depression. Overall, this study may provide preliminary evidence for the early diagnosis and treatment of depression using urinary VOCs.

Depression is one of the key diseases covered by the World Health Organization’s Mental Health Gap Action Programme. It is estimated that approximately 3.8% of the global population is affected by depression.^[28,29]^ In the United States, mental illnesses result in annual economic losses amounting to billions of dollars. The international community has jointly invested substantial resources to improve mental health. Consequently, the precise detection of modifiable risk factors for depression represents a critical public health priority.^[30]^ Exposure to air pollution is associated with many diseases.^[31–33]^ Accumulating evidence indicates that airborne pollutant exposure exerts detrimental effects on psychiatric health, particularly elevating susceptibility to major depressive disorder and anxiety syndromes.^[34,35]^ Exposure to VOCs, as a constituent of airborne pollution, may be associated with the onset and progression of depression.

VOCs are ubiquitously encountered in routine human activities, exemplified by automobile exhaust emissions, cooking practices, wood burning, industrial operations, tobacco consumption, cleaning agents, building materials, and household items.^[36,37]^ Accumulating evidence demonstrates that exposure to VOCs constitutes a significant modifiable risk factor for population health deterioration.^[38]^ In the human body, volatile organic compounds are metabolized into a series of hydroxyl ring-opening compounds in the liver under the action of hepatic cytochrome P450, and these compounds can be excreted through urine.^[39,40]^ Detecting urinary VOC metabolites can better reflect the endocrine status of VOCs. Since the average half-life of most VOC compounds is <10 hours, urinary VOC metabolites more often represent short-term rather than long-term VOC exposure levels.^[41]^ Because VOCs are volatile, directly detecting their content in the blood does not yield accurate results. Consequently, urinary concentrations of water-soluble VOC metabolites may serve as a robust biomarker for quantifying the VOC exposure index.^[42]^ Epidemiological evidence highlights urinary VOC exposure as a modifiable risk factor for various disease outcomes.^[43,44]^ Recently, multiple studies have shown that there are associations between urinary VOC metabolites and diseases such as depression, obesity, and chronic respiratory diseases.^[8,45,46]^ Kilburn et al reported that the proportion of people with depression was significantly higher in the group exposed to VOCs compared with those not exposed to VOCs.^[47]^ In addition, an analysis of 3240 participants using the NHANES database showed that the exposure levels of multiple urinary VOC metabolites were significantly and positively correlated with the risk of depression.^[15]^ The study, through multiple linear and logistic regression models, found that among the 16 analyzed mVOCs, 10 were positively associated with depression, indicating that both single and mixed VOC exposures may have a negative impact on mental health.^[15]^ Similarly, in this study, URXCYM, URXGAM, URXMAD, and URXPHG were positively correlated with the risk of depression. Additionally, the results of the WQS regression showed that URXCYM had the highest weight (70.46%), followed by URXPHG (12.29%) and URXMAD (10.23%). Collectively, extant epidemiological evidence and current findings demonstrate that exposure to VOCs contributes to an elevated risk of depression, highlighting the need for increased public health attention to the multifaceted impact of VOCs on mental health outcomes.

CIAD represents a major contributor to the global burden of disease, significantly impacting both disability-adjusted life years and mortality rates worldwide. The pathophysiological processes of these diseases are complex, involving the interaction between genetics and multiple environmental factors, among which chronic airway inflammation is the core feature.^[16,17]^ Oxidative stress and systemic inflammatory responses represent potential mechanistic pathways underlying the association between VOC exposure and CIAD pathogenesis. Oxidative DNA damage, inflammatory responses, and pulmonary epithelial injury collectively mediate the adverse effects of individual VOC metabolites on respiratory functional decline.^[48,49]^ Evidence indicates that specific VOC metabolite mixtures exacerbate chronic bronchitis and emphysema pathology through inflammatory cascade activation.^[50]^ Epidemiological assessments across diverse populations have characterized the effects of individual VOC metabolites on CIAD progression and pulmonary function decline. Epidemiological analyses leveraging the NHANES have identified statistically significant linkages between over ten VOC metabolites and both COPD and impaired pulmonary function.^[51–53]^ Epidemiological data demonstrate that urinary metabolites of dimethylformamide, acrolein, and 1-bromopropane exhibit significant negative correlations with pulmonary function parameters.^[49,54]^ In addition, CIAD severely impairs patients’ mental health and quality of life through persistent symptoms, functional limitations, and uncertainty about the future. The dyspnea, sleep disorders, and activity limitations caused by the disease itself directly lead to negative emotional experiences among patients.^[55]^ This may be due to the impact of systemic inflammation on the structure and function of specific neural networks, especially the brain regions responsible for processing internal somatic signals (interoception) and emotional regulation. Mendelian randomization analysis identified the intermediary function of the interoceptive neural circuit (INC) in mediating the progression from somatic conditions such as asthma to comorbid depressive states.^[56]^ The study found that various somatic diseases can cause morphological changes in the INC, and these morphological changes in the INC increase the risk of depression, confirming the key hub position of L-VDC in the “lung–brain” connection.^[56]^ Consistent with our findings, CIAD was established as a significant mediating factor in the association between VOCs and depression.

This investigation acknowledges several methodological constraints. Primarily, the quantified VOC metabolites capture short-term exposure windows, whereas depression pathogenesis involves chronic progressive mechanisms. Confounding effects may arise from chronic alterations in these metabolites. The cross-sectional design of this investigation constrains causal inference regarding associations between complex VOC metabolite mixtures and depression etiology. Methodological limitations inherent in this investigation include systematic measurement errors such as information bias. Secondly, additional depression risk factors, including airborne pollutant exposure, occupational hazards, and dietary patterns, demonstrate epidemiological linkages with VOC exposure pathways. Therefore, although we conducted statistical adjustments to control potential confounding factors, residual confounding from unmeasured covariates cannot be excluded. Finally, while individual VOC effects on depression have been documented across diverse regional populations, the combinatorial impact of VOC metabolites remains poorly characterized, with existing evidence predominantly derived from the NHANES dataset. Additional research is essential for external validation of their combined effects and exploration of the contributions made by individual components among other populations.

## 5. Conclusion

In the NHANES data, an association was found between urinary VOCs and an increased risk of depression. CIAD plays a certain role in the correlation between urinary VOCs and depression. Moreover, urinary VOCs can be used to predict the risk of depression. More research is needed to clarify the mechanisms underlying the link between urinary VOCs and depression and their clinical application value.

## Author contributions

**Conceptualization:** Jingshan Bai, Qian Huang, Wenqiang Li, Zhiping Deng, Quan Yuan.

**Data curation:** Jingshan Bai, Qian Huang, Ying Yang, Zhiping Deng, Quan Yuan.

**Formal analysis:** Jingshan Bai.

**Funding acquisition:** Ying Yang, Zhiping Deng, Quan Yuan.

**Methodology:** Jingshan Bai, Qian Huang.

**Project administration:** Wenqiang Li, Zhiping Deng, Quan Yuan.

**Resources:** Ying Yang, Zhiping Deng, Quan Yuan.

**Software:** Jingshan Bai, Qian Huang, Wenqiang Li.

**Supervision:** Ying Yang, Zhiping Deng, Quan Yuan.

**Visualization:** Jingshan Bai, Wenqiang Li.

**Writing – original draft:** Jingshan Bai, Qian Huang, Wenqiang Li, Ying Yang, Zhiping Deng.

**Writing – review & editing:** Quan Yuan.

## Supplementary Material



## References

[1] Zhu Y Ju Y Wang M Yang Y Wu R. Association of volatile organic compounds exposure with the risk of depression in U.S. adults: a cross‑sectional study from NHANES 2013–2016. Int Arch Occup Environ Health. 2023;96:1101–11. 37368145 10.1007/s00420-023-01993-6

[2] Fogarty AS Proudfoot J Whittle EL. Preliminary evaluation of a brief web and mobile phone intervention for men with depression: men’s positive coping strategies and associated depression, resilience, and work and social functioning. JMIR Ment Health. 2017;4:e33. 28798009 10.2196/mental.7769 PMC5571234

[3] Meng R Yu C Liu N. Association of depression with all-cause and cardiovascular disease mortality among adults in China. JAMA Netw Open. 2020;3:e1921043. 32049295 10.1001/jamanetworkopen.2019.21043 PMC7212017

[4] Planchez B Surget A Belzung C. Animal models of major depression: drawbacks and challenges. J Neural Transm (Vienna). 2019;126:1383–408. 31584111 10.1007/s00702-019-02084-y PMC6815270

[5] Herrman H Kieling C McGorry P Horton R Sargent J Patel V. Reducing the global burden of depression: a Lancet-World Psychiatric Association Commission. Lancet. 2019;393:e42–3. 30482607 10.1016/S0140-6736(18)32408-5

[6] Deuschl G Beghi E Fazekas F. The burden of neurological diseases in Europe: an analysis for the Global Burden of Disease Study 2017. Lancet Public Health. 2020;5:e551–67. 33007212 10.1016/S2468-2667(20)30190-0

[7] Jacobson MH Ghassabian A Gore AC Trasande L. Exposure to environmental chemicals and perinatal psychopathology. Biochem Pharmacol. 2022;195:114835. 34774531 10.1016/j.bcp.2021.114835 PMC8712457

[8] Wu M Liu M Zhang Y. Serum HDL partially mediates the association between exposure to volatile organic compounds and kidney stones: a nationally representative cross-sectional study from NHANES. Sci Total Environ. 2024;907:167915. 37858818 10.1016/j.scitotenv.2023.167915

[9] Zhou X Zhou X Wang C Zhou H. Environmental and human health impacts of volatile organic compounds: a perspective review. Chemosphere. 2023;313:137489. 36513206 10.1016/j.chemosphere.2022.137489

[10] Daouda M Carforo A Jack D Hernández D. Correspondence on “Home is where the pipeline ends: characterization of volatile organic compounds present in natural gas at the point of the residential end user.”. Environ Sci Technol. 2023;57:1848–9. 36657100 10.1021/acs.est.2c09423 PMC11888120

[11] Pandit GG Srivastava PK Rao AM. Monitoring of indoor volatile organic compounds and polycyclic aromatic hydrocarbons arising from kerosene cooking fuel. Sci Total Environ. 2001;279:159–65. 11712593 10.1016/s0048-9697(01)00763-x

[12] Wei Y Zhu J. Para-Dichlorobenzene exposure is associated with thyroid dysfunction in US adolescents. J Pediatr. 2016;177:238–43. 27476635 10.1016/j.jpeds.2016.06.085

[13] Ye D Klein M Chang HH. Estimating acute cardiorespiratory effects of ambient volatile organic compounds. Epidemiology. 2017;28:197–206. 27984424 10.1097/EDE.0000000000000607 PMC5285400

[14] Yu SY Koh EJ Kim SH. Integrated analysis of multi-omics data on epigenetic changes caused by combined exposure to environmental hazards. Environ Toxicol. 2021;36:1001–10. 33438815 10.1002/tox.23099

[15] Ma T Wang X He W. Expose to volatile organic compounds is associated with increased risk of depression: a cross-sectional study. J Affect Disord. 2024;363:239–48. 39038625 10.1016/j.jad.2024.07.028

[16] Nalini M Poustchi H Bhandari D. Exposure to volatile organic compounds and chronic respiratory disease mortality, a case-cohort study. Respir Res. 2025;26:88. 40045272 10.1186/s12931-025-03165-1 PMC11884121

[17] Rupani H Fong WCG Kyyaly A Kurukulaaratchy RJ. Recent insights into the management of inflammation in asthma. J Inflamm Res. 2021;14:4371–97. 34511973 10.2147/JIR.S295038 PMC8421249

[18] Xiong Y Liu X Li T. The urinary metabolites of volatile organic compounds and asthma in young children: NHANES 2011–2018. Heliyon. 2024;10:e24199. 38317969 10.1016/j.heliyon.2024.e24199 PMC10838696

[19] Park D Ha EK Jung H. Associations of personal urinary volatile organic compounds and lung function in children. J Asthma. 2024;61:801–7. 38198535 10.1080/02770903.2024.2303770

[20] Xie S Huang J Chen G. Urine volatile organic compounds in predicting chronic obstructive pulmonary disease risk in a national observational study. Environ Sci Process Impacts. 2025;27:2588–600. 40704434 10.1039/d5em00181a

[21] Jimenez-Peinado A Laguna-Munoz D Jaen-Moreno MJ. Respiratory disease in people with major depressive disorder: a systematic review and Meta-analysis. Eur Psychiatry. 2025;68:e34. 39904730 10.1192/j.eurpsy.2025.13 PMC11883783

[22] Xu X Li S Chen Y. Association between allergic diseases and mental health conditions: an umbrella review. J Allergy Clin Immunol. 2025;155:701–13. 39521284 10.1016/j.jaci.2024.10.030

[23] Mallya SG Upadhyay A Psoter KJ. Association between difficulty affording medications and outcomes in chronic obstructive pulmonary disease. Ann Am Thorac Soc. 2025;22:984–91. 39970425 10.1513/AnnalsATS.202410-1020OC PMC12254152

[24] Luo Z Chen P Chen S Kong X Ma H Cao C. Relationship between advanced lung cancer inflammation index and all-cause and cause-specific mortality among chronic inflammatory airway diseases patients: a population-based study. Front Immunol. 2025;16:1585927. 40443682 10.3389/fimmu.2025.1585927 PMC12119279

[25] Fu Q Wu Y Zhu M. Identifying cardiovascular disease risk in the U.S. population using environmental volatile organic compounds exposure: a machine learning predictive model based on the SHAP methodology. Ecotoxicol Environ Saf. 2024;286:117210. 39447292 10.1016/j.ecoenv.2024.117210

[26] Lamers F Jonkers CC Bosma H Penninx BWJH Knottnerus JA van Eijk JTM. Summed score of the Patient Health Questionnaire-9 was a reliable and valid method for depression screening in chronically ill elderly patients. J Clin Epidemiol. 2008;61:679–87. 18538262 10.1016/j.jclinepi.2007.07.018

[27] Yan Z Xu Y Li K Liu L. Association between high-density lipoprotein cholesterol and type 2 diabetes mellitus: dual evidence from NHANES database and Mendelian randomization analysis. Front Endocrinol (Lausanne). 2024;15:1272314. 38455653 10.3389/fendo.2024.1272314 PMC10917910

[28] Avan A Hachinski V. Global, regional, and national trends of dementia incidence and risk factors, 1990–2019: a Global Burden of Disease study. Alzheimers Dement. 2023;19:1281–91. 36044376 10.1002/alz.12764

[29] Evans-Lacko S Aguilar-Gaxiola S Al-Hamzawi A. Socio-economic variations in the mental health treatment gap for people with anxiety, mood, and substance use disorders: results from the WHO World Mental Health (WMH) surveys. Psychol Med. 2018;48:1560–71. 29173244 10.1017/S0033291717003336 PMC6878971

[30] Kioumourtzoglou MA. Identifying modifiable risk factors of mental health disorders-the importance of urban environmental exposures. JAMA Psychiatry. 2019;76:569–70. 30916721 10.1001/jamapsychiatry.2019.0010

[31] Hanna GB Boshier PR Markar SR Romano A. Accuracy and methodologic challenges of volatile organic compound-based exhaled breath tests for cancer diagnosis: a systematic review and meta-analysis. JAMA Oncol. 2019;5:e182815. 30128487 10.1001/jamaoncol.2018.2815 PMC6439770

[32] Lei T Qian H Yang J Hu Y. The association analysis between exposure to volatile organic chemicals and obesity in the general USA population: a cross-sectional study from NHANES program. Chemosphere. 2023;315:137738. 36608892 10.1016/j.chemosphere.2023.137738

[33] Wang R Ye C Huang X. cMIND Diet, indoor air pollution, and depression: a cohort study based on the CLHLS from 2011 to 2018. Nutrients. 2023;15:1203. 36904202 10.3390/nu15051203 PMC10005708

[34] Tan J Chen N Bai J. Ambient air pollution and the health-related quality of life of older adults: evidence from Shandong China. J Environ Manage. 2023;336:117619. 36924708 10.1016/j.jenvman.2023.117619

[35] Trombley J. Fine particulate matter exposure and pediatric mental health outcomes: an integrative review. J Nurs Scholarsh. 2023;55:977–1007. 36941765 10.1111/jnu.12888

[36] Konkle SL Zierold KM Taylor KC Riggs DW Bhatnagar A. National secular trends in ambient air volatile organic compound levels and biomarkers of exposure in the United States. Environ Res. 2020;182:108991. 31835113 10.1016/j.envres.2019.108991 PMC7294699

[37] Su FC Jia C Batterman S. Extreme value analyses of VOC exposures and risks: a comparison of RIOPA and NHANES datasets. Atmos Environ (1994). 2012;62:97–106. 25705112 10.1016/j.atmosenv.2012.06.038 PMC4334151

[38] Wei C Chen Y Yang Y. Assessing volatile organic compounds exposure and prostate-specific antigen: National Health and Nutrition Examination Survey, 2001–2010. Front Public Health. 2022;10:957069. 35968491 10.3389/fpubh.2022.957069 PMC9372286

[39] Rooij BMD Commandeur JNM Vermeulen NPE. Mercapturic acids as biomarkers of exposure to electrophilic chemicals:applications to environmental and industrial chemicals. Biomarkers. 1998;3:239–303. 23899357 10.1080/135475098231101

[40] Hu Y Niu Z Cao C. Volatile organic compounds (VOC) metabolites in urine are associated with increased systemic inflammation levels, and smokers are identified as a vulnerable population. Ecotoxicol Environ Saf. 2024;288:117398. 39612684 10.1016/j.ecoenv.2024.117398

[41] Ashley DL Zhu W Bhandari D. Influence of half-life and smoking/nonsmoking ratio on biomarker consistency between waves 1 and 2 of the population assessment of tobacco and health study. Cancer Epidemiol Biomarkers Prev. 2024;33:80–7. 37823832 10.1158/1055-9965.EPI-23-0538 PMC10843274

[42] Yang Y Lin M Tang J Ma S Yu Y. Derivatization gas chromatography negative chemical ionization mass spectrometry for the analysis of trace organic pollutants and their metabolites in human biological samples. Anal Bioanal Chem. 2020;412:6679–90. 32556566 10.1007/s00216-020-02762-x

[43] Wang S Luo J Zhang F. Association between blood volatile organic aromatic compound concentrations and hearing loss in US adults. BMC Public Health. 2024;24:623. 38413886 10.1186/s12889-024-18065-0 PMC10897984

[44] Zhou HL Di DS Cui ZB. Whole-body aging mediates the association between exposure to volatile organic compounds and osteoarthritis among U.S. middle-to-old-aged adults. Sci Total Environ. 2024;907:167728. 37827324 10.1016/j.scitotenv.2023.167728

[45] Lv JJ Li XY Shen YC. Assessing volatile organic compounds exposure and chronic obstructive pulmonary diseases in US adults. Front Public Health. 2023;11:1210136. 37475768 10.3389/fpubh.2023.1210136 PMC10354632

[46] Tang L Liu M Tian J. Volatile organic compounds exposure associated with depression among U.S. adults: results from NHANES 2011–2020. Chemosphere. 2024;349:140690. 37995973 10.1016/j.chemosphere.2023.140690

[47] Kilburn KH Warshaw RH. Neurobehavioral testing of subjects exposed residentially to groundwater contaminated from an aluminum die-casting plant and local referents. J Toxicol Environ Health. 1993;39:483–96. 8345533 10.1080/15287399309531766

[48] Wang B Fan L Yang S. Cross-sectional and longitudinal relationships between urinary 1-bromopropane metabolite and pulmonary function and underlying role of oxidative damage among urban adults in the Wuhan-Zhuhai cohort in China. Environ Pollut. 2022;313:120147. 36096263 10.1016/j.envpol.2022.120147

[49] Wang B Yu L Liu W. Cross-sectional and longitudinal associations of acrolein exposure with pulmonary function alteration: assessing the potential roles of oxidative DNA damage, inflammation, and pulmonary epithelium injury in a general adult population. Environ Int. 2022;167:107401. 35850081 10.1016/j.envint.2022.107401

[50] Wang Y Yu Y Zhang X. Combined association of urinary volatile organic compounds with chronic bronchitis and emphysema among adults in NHANES 2011–2014: the mediating role of inflammation. Chemosphere. 2024;361:141485. 38438022 10.1016/j.chemosphere.2024.141485

[51] Huang Q Li S Wan J Nan W He B. Association between ethylene oxide exposure and prevalence of COPD: evidence from NHANES 2013–2016. Sci Total Environ. 2023;885:163871. 37149189 10.1016/j.scitotenv.2023.163871

[52] Mendy A Burcham S Merianos AL. Urinary volatile organic compound metabolites and reduced lung function in U.S. adults. Respir Med. 2022;205:107053. 36399896 10.1016/j.rmed.2022.107053 PMC9869342

[53] Wang Y Han X Li J. Associations between the compositional patterns of blood volatile organic compounds and chronic respiratory diseases and ages at onset in NHANES 2003–2012. Chemosphere. 2023;327:138425. 36931402 10.1016/j.chemosphere.2023.138425

[54] Wang B Yang S Guo Y. Association of urinary dimethylformamide metabolite with lung function decline: the potential mediating role of systematic inflammation estimated by C-reactive protein. Sci Total Environ. 2020;726:138604. 32305772 10.1016/j.scitotenv.2020.138604

[55] An TJ Lim J Lee H. Breathlessness, frailty, and sarcopenia in older adults. Chest. 2024;166:1476–86. 39209061 10.1016/j.chest.2024.07.180

[56] Zhou H Jiang C Jiang W Zhou Z Yuan Y. Interoceptive neural circuits mediating the progression from somatic diseases to comorbid depression. Psychother Psychosom. 2025;25:1–19. 10.1159/000547584 40716430

