# Supplementary material for: Mediating role of chronic inflammatory airway diseases in the association between volatile organic compounds exposure and depression
Source: Medicine (Baltimore). 2026 Apr 24;105(17):e48522. doi: 10.1097/MD.0000000000048522 (PMC13124432; doi:10.1097/MD.0000000000048522)
Supplement: Supplementary file 1 [file medi-105-e48522-s001.pdf]

Supplementary Table1. List of urine VOCs included in this study.

Varible	Analyte	Code name
URXAAM	N-Acetyl-S-(2-carbamoylethyl)-L-cysteine	A
URXAMC	N-Acetyl-S-(N-methylcarbamoyl)-L-cysteine	B
URXATC	2-Aminothiazoline-4-carboxylic acid	C
URXCEM	N-Acetyl-S-(2-carboxyethyl)-L-cysteine	D
URXCYM	N-Acetyl-S-(2-cyanoethyl)-L-cysteine	E
URXDHB	N-Acetyl-S-(3,4-dihydroxybutyl)-L-cysteine	F
URXGAM	N-Acetyl-S-(2-carbamoyl-2-hydroxyethyl)-L-cysteine	G
URXHEM	N-Acetyl-S-(2-hydroxyethyl)-L-cysteine	H
URXHPM	N-Acetyl-S-(3-hydroxypropyl)-L-cysteine	I
URXHP2	N-Acetyl-S-(2-hydroxypropyl)-L-cysteine	J
URXPMM	N-Acetyl-S-(3-hydroxypropyl-1-methyl)-L-cysteine	K
URXMAD	Mandelic acid	L
URX2MH	2-Methylhippuric acid	M
URX34M	3- and 4-Methylhippuric acid	N
URXMB3	N-Acetyl-S-(4-hydroxy-2-butenyl)-L-cysteine	O
URXPHG	Phenylglyoxylic acid	P
